# Association Between Aneurysmal Hemodynamics and Rupture Risk of Unruptured Intracranial Aneurysms

**DOI:** 10.3389/fneur.2022.818335

**Published:** 2022-04-21

**Authors:** Zhongbin Tian, Xifeng Li, Chao Wang, Xin Feng, Kaijian Sun, Yi Tu, Hengxian Su, Xinjian Yang, Chuanzhi Duan

**Affiliations:** ^1^National Key Clinical Specialty, Engineering Technology Research Center of Education Ministry of China, Guangdong Provincial Key Laboratory on Brain Function Repair and Regeneration, Neurosurgery Institute, Department of Neurosurgery, Zhujiang Hospital, Southern Medical University, Guangzhou, China; ^2^Department of Neurosurgery, Binzhou Medical University Hospital, Binzhou, China; ^3^Department of Interventional Neuroradiology, Beijing Neurosurgical Institute and Beijing Tian Tan Hospital, Capital Medical University, Beijing, China

**Keywords:** hemodynamics, rupture risk, PHASES score, unruptured intracranial aneurysm, wall shear stress

## Abstract

**Background:**

Assessing rupture risk in patients with unruptured intracranial aneurysms (UIAs) remains challenging. Hemodynamics plays an important role in the natural history of intracranial aneurysms. This study aimed to compare aneurysmal hemodynamic features between patients with different rupture risk as determined by PHASES score.

**Methods:**

We retrospectively examined 238 patients who harbored a solitary saccular UIA. Patients were stratified by rupture risk into low-, intermediate-, and high-risk groups according to PHASES score. Flow simulations were performed to compare differences in hemodynamics among the groups.

**Results:**

Aneurysmal time-averaged wall shear stress (WSSa) and normalized WSS (WSSn) decreased progressively as PHASES score increased. WSSa and WSSn significantly differed among the low-, intermediate-, and high-risk groups (*p* < 0.001). WSSa was significantly lower in the high-risk group than the low-risk group (*p* < 0.001) and the intermediate-risk group (*p* = 0.004). WSSn was also significantly lower in the high-risk group than the low-risk group (*p* < 0.001) and the intermediate-risk group (*p* = 0.001).

**Conclusions:**

Low WSS was significantly associated with higher risk of intracranial aneurysm rupture as determined by PHASES score, indicating that hemodynamics may play an important role in aneurysmal rupture. In the future, a multidimensional rupture risk prediction model that includes hemodynamic parameters should be investigated.

## Introduction

Unruptured intracranial aneurysms (UIAs) occur in approximately 3% to 8% of the general population (1, 2). Their prevalence is rising because of advances in intracranial diagnostic imaging. Subarachnoid hemorrhage after intracranial aneurysm rupture is associated with high mortality and morbidity (3, 4). However, decision making regarding UIA treatment remains challenging and must consider the balance between the risks of rupture and the risks of treatment (5). Assessment of rupture risk can assist the decision-making process by identifying UIAs prone to rupture.

Previous studies have attempted to identify factors related to aneurysmal rupture by comparing morphologic and hemodynamic characteristics between ruptured and unruptured aneurysms (6–8). Hemodynamic features appear to play a key role, particularly wall shear stress (WSS). However, findings based on direct comparison of ruptured and unruptured aneurysms may be invalid, as aneurysmal morphology changes after rupture (9). The relationship between aneurysmal hemodynamics and rupture requires further investigation.

The Population, Hypertension, Age, Size of aneurysm, Earlier subarachnoid hemorrhage, Site of aneurysm (PHASES) score was developed in 2014 to assess aneurysm rupture risk in patients with incidentally detected aneurysms. This score predicts the 5-year risk of rupture based on 6 risk factors (population, hypertension, age, size of aneurysm, earlier subarachnoid hemorrhage from another aneurysm, site of aneurysm) (10); hemodynamic factors are not considered. Therefore, this study aimed to compare aneurysmal hemodynamic characteristics between patients with different rupture risk as determined by PHASES score.

## Methods

### Patient Selection

This retrospective study was approved by the institutional review board of our hospital. Written informed consent was obtained from all patients or their family members. Two hundred thirty-eight patients with a solitary unruptured saccular cerebral aneurysm who underwent angiography from August 2016 to July 2019 were enrolled. We excluded patients with fusiform or dissecting aneurysms, multiple aneurysms, ruptured aneurysms, and those with unsatisfactory three-dimensional aneurysm imaging for the hemodynamics simulation. Patient and aneurysmal characteristics including age, gender, hypertension, and aneurysm size and location were recorded.

### Hemodynamics Simulation

Computed fluid dynamic numerical simulation was performed as described previously (11). Briefly, three-dimensional patient-specific aneurysm models were reconstructed from rotational angiography images. Each aneurysm model was imported into ICEM CFD software (ANSYS Inc., Canonsburg, PA, USA) to create approximately 3 million finite volume tetrahedral elements; the largest element was 0.2 mm. Then, CFX V.14.0 software (ANSYS, Inc.) was used to simulate blood hemodynamics. The governing equations underlying the calculation were the Navier–Stokes equations. Blood was assumed as an incompressible Newtonian fluid with a density of 1,060 kg/m^3^ and a viscosity of 0.004 kg/m/s. The average Reynolds number was within the range of normal blood flow in human cerebral arteries. The vessel wall was Assumed to be rigid with no-slip boundary conditions. The inflow boundary condition was a pulsatile period velocity profile of a normal subject. Three cardiac cycle simulations were performed for numerical stability and the results of the last cardiac cycle was recorded. After hemodynamics simulation, the time-averaged wall shear stress (WSSa) was calculated in each patient by integrating the WSS magnitude over the cardiac cycle. Then, the WSSa on the aneurysm was normalized by the average parent vessel WSS to obtain the normalized WSS (WSSn).

### PHASES Score

The PHASES score was calculated for each patient to predict the 5-year absolute risk of aneurysm rupture. All patients included in this study were Chinese and scored zero points for geographical region (12, 13). Patients were divided into 3 groups according to rupture risk as determined by PHASES score: low-risk (0–4 points), intermediate-risk (5–9 points), and high-risk (≥10 points). The corresponding predicted 5-year rupture risk for the low-, intermediate-, and high-risk groups is <1.3, 1.3–5.3, and ≥5.3%, respectively.

### Statistical Analysis

Statistical analyses were performed using SPSS software version 17.0 (IBM Corp., Armonk, NY, USA). Continuous data are expressed as means with standard deviation. Categorical data are expressed as numbers with percentage. The Kruskal–Wallis test with *post hoc* Bonferroni correction was used to analyze the hemodynamic differences among groups. *P* < 0.05 was considered significant.

## Results

### Patient and Aneurysm Characteristics

Patient and aneurysm characteristics are shown in Table 1. The mean age of the 238 patients was 54.7 ± 9.4 years. Of the 238 patients, 160 (67.2%) were women. The number of patients in the low-, intermediate-, and high-risk groups was 140, 83, and 15, respectively.

**Table 1:** Patient and aneurysm characteristics.

	**Low-risk group (*n* = 140)**	**Intermediate-risk group (*n* = 83)**	**High-risk group (*n* = 15)**	***P*** **-value**	**Total (*n* = 238)**
Age (year)	54.9 ± 8.6	55.4 ± 10.0	48.8 ± 11.4		54.7 ± 9.4
<70	136 (97.1)	77 (92.8)	15 (100.0)	0.204	228 (95.8)
≥70	4 (2.9)	6 (7.2)	0 (0)		10 (4.2)
Gender (%)				0.199	
Male	40 (28.6)	31 (37.3)	7 (46.7)		78 (32.8)
Female	100 (71.4)	52 (62.7)	8 (53.3)		160 (67.2)
Aneurysm size (mm)	5.3 ± 1.6	9.9 ± 4.4	19.6 ± 4.3		7.8 ± 4.8
<7.0	121 (86.4)	19 (22.9)	0 (0)	<0.001	140 (58.8)
7.0–9.9	19 (13.6)	22 (26.5)	0 (0)		41 (17.2)
10.0–19.9	0 (0)	42 (50.6)	5 (33.3)		47 (19.7)
≥20	0 (0)	0 (0)	10 (66.7)		10 (4.2)
Location (%)				<0.001	
ICA	113 (80.7)	40 (48.2)	9 (60.0)		162 (68.1)
MCA	15 (10.7)	8 (9.6)	0 (0)		23 (9.7)
ACA/Pcom/Posterior	12 (8.6)	35 (42.2)	6 (40.0)		53 (22.3)

*ICA, internal carotid artery; MCA, middle cerebral artery; ACA, anterior cerebral artery; PComA, posterior communicating artery*.

### Hemodynamic Analysis

**Figure 1:**
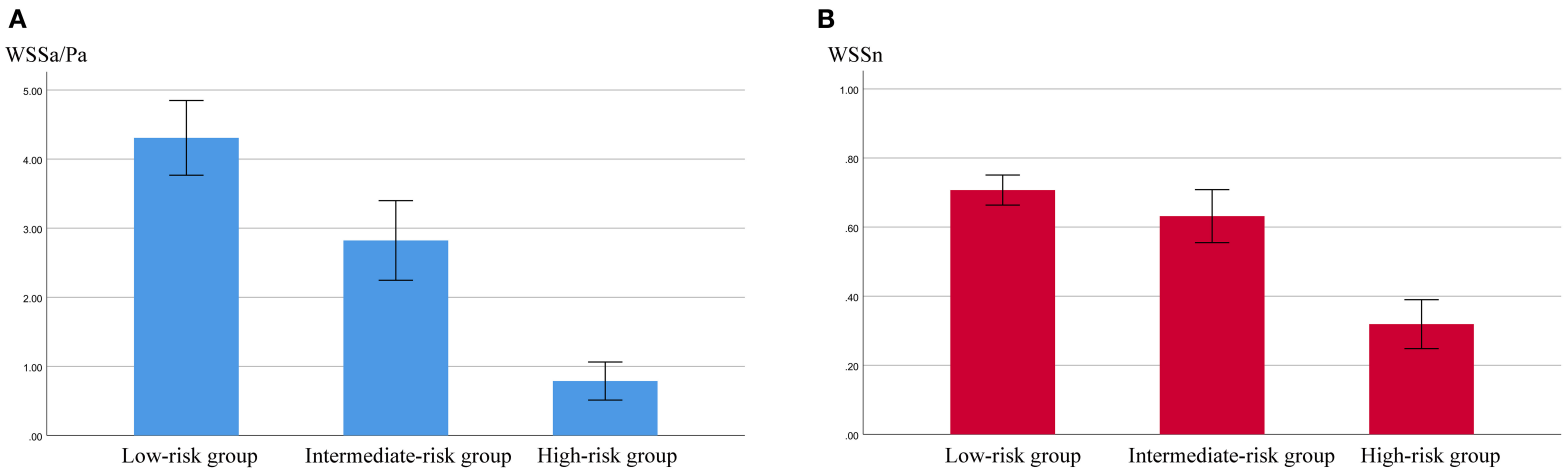
The values of aneurysmal time-averaged wall shear stress (WSSa) **(A)** and normalized WSS (WSSn) **(B)** in the low-, intermediate-, and high-risk groups.

The results of the hemodynamic analyses are shown in Tables 2, 3. WSSa in the low-, intermediate-, and high-risk groups was 4.31 ± 3.20, 2.82 ± 2.62, and 0.79 ± 0.53 Pa; the corresponding WSSn values were 0.71 ± 0.26, 0.63 ± 0.35, and 0.32 ± 0.14, respectively. As shown in Figure 1, WSSa and WSSn values progressively decreased as PHASES score increased. WSSa and WSSn significantly differed among the groups (*p* < 0.001).

**Table 2:** Aneurysmal hemodynamics in the low-, intermediate-, and high-risk groups.

	**Low-risk group**	**Intermediate-risk group**	**High-risk group**	***p*** **-value**
WSSa (Pa)	4.31 ± 3.20	2.82 ± 2.62	0.79 ± 0.53	*p* < 0.001
WSSn	0.71 ± 0.26	0.63 ± 0.35	0.32 ± 0.14	*p* < 0.001

*WSSa, time-averaged wall shear stress; WSSn, normalized WSSa by the average parent vessel WSS*.

**Table 3:** Multiple comparisons of aneurysmal wall shear stress among low-, intermediate-, and high-risk groups.

	**WSSa (Pa)**	**Adjusted *p*-value**	**WSSn**	**Adjusted *p*-value**
Low-risk group vs. Intermediate-risk group	4.31 ± 3.20 vs. 2.82 ± 2.62	<0.001	0.71 ± 0.26 vs. 0.63 ± 0.35	0.068
Low-risk group vs. High-risk group	4.31 ± 3.20 vs. 0.79 ± 0.53	<0.001	0.71 ± 0.26 vs. 0.32 ± 0.14	<0.001
Intermediate-risk group vs. High-risk group	2.82 ± 2.62 vs. 0.79 ± 0.53	0.004	0.63 ± 0.35 vs. 0.32 ± 0.14	0.001

*WSSa, time-averaged wall shear stress; WSSn, normalized WSSa by the average parent vessel WSS; adjusted p-value, p-value adjusted by Bonferroni correction*.

**Figure 2:**
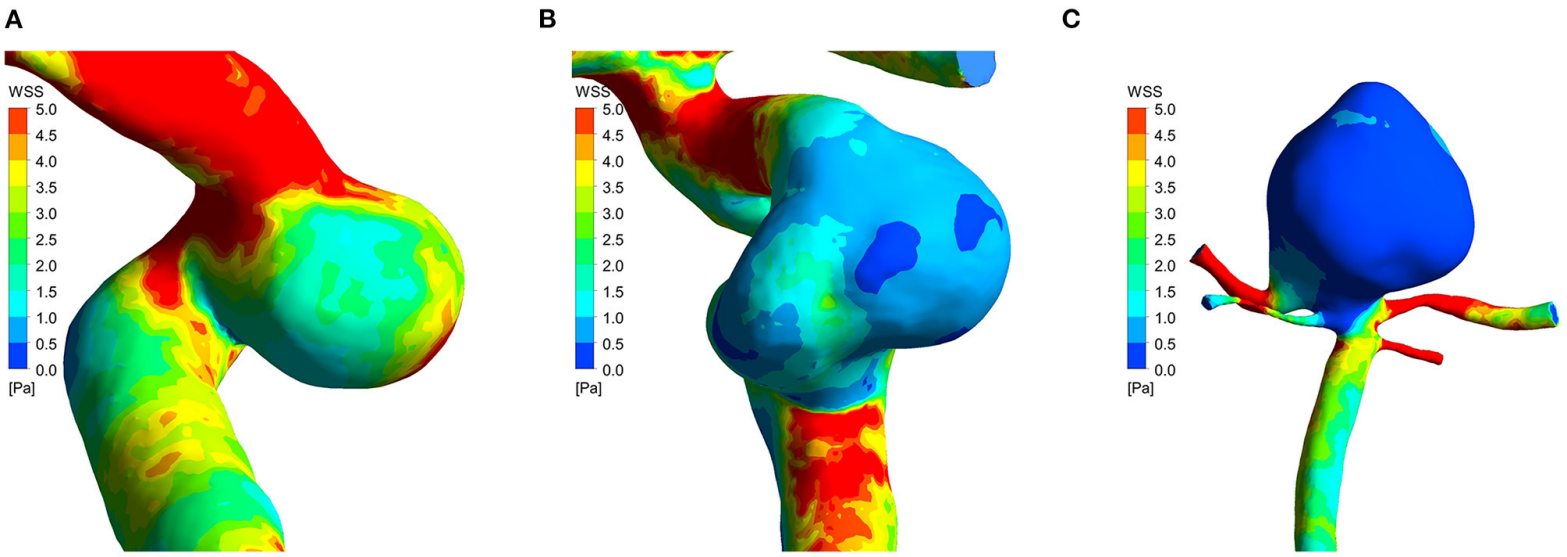
Time-averaged wall shear stress distribution maps of three cases in low-risk group **(A)**, intermediate-risk group **(B)**, and high-risk group **(C)**. PHASES score of the three cases was 3, 6, and 11, respectively. The time-averaged wall shear stress progressively decreased as aneurysm rupture risk increased.

Analysis after *post hoc* Bonferroni correction showed that WSSa was significantly lower in the high-risk group than the low-risk group (*p* < 0.001) and the intermediate-risk group (*p* = 0.004). Additionally, WSSa was significantly lower in the intermediate-risk group than the low-risk group (*p* < 0.001; Figure 2). Similarly, WSSn was significantly lower in the high-risk group than the low-risk group (*p* < 0.001) and the intermediate-risk group (*p* = 0.001). Although WSSn was lower in the intermediate-risk group than the low-risk group, the difference was not significant (*p* = 0.068).

## Discussion

The decision to treat an UIA remains challenging and must balance the risk of aneurysmal rupture and the risks of treatment. Identifying patient and aneurysmal factors associated with higher rupture risk can assist physicians in treatment decision making. In this study, we compared aneurysmal hemodynamics between patients with different risks of rupture as determined by PHASES score. We found that aneurysmal WSS decreased significantly as the PHASES score increased. Our findings may improve the ability to predict UIA rupture and increase understanding of the mechanism of aneurysmal rupture.

A pure natural history study about aneurysm rupture is difficult to undertake due to the catastrophic results associated with aneurysm rupture, and the number of aneurysm ruptures during follow-up was too small for valid analyses. Previous studies have usually investigated the role of hemodynamics in aneurysmal rupture using ruptured aneurysms (6–8). However, aneurysm shape may change after rupture, which can alter aneurysmal hemodynamic features. Therefore, hemodynamic comparisons between unruptured and ruptured aneurysms may be invalid. Kono et al. analyzed the hemodynamics of an aneurysm that ruptured soon after imaging and found that WSS changed by 20–30% after rupture (14). Similarly, Wang et al. also found altered aneurysmal morphology and a 30% change in WSS after aneurysm rupture (15). Hemodynamic features that might predict rupture should ideally be investigated before aneurysmal rupture.

Few studies have investigated aneurysmal hemodynamic features just before rupture and most have been case reports (16, 17). Two different case-control studies that examined hemodynamic features before rupture have reported that low WSS is a predictor of aneurysm rupture (18, 19). However, both had a very small sample size and included only internal carotid artery aneurysms; therefore, their evidence remains weak. In our study, the sample size was large and patients were stratified according to aneurysm rupture risk as determined by PHASES score. Consistent with previous studies, we found that WSS significantly decreased as PHASES score increased and that WSS was significantly lower in aneurysms with a higher risk of rupture.

Hemodynamics plays an important role in aneurysmal rupture and WSS is the most studied hemodynamic parameter (7, 20). WSS is defined as the tangential drag force per unit area of endothelial surface (21) and is transduced through endothelial cell mechanoreceptors into biological signals that can regulate gene expression, endothelial cell function, and blood vessel structure (22). Low WSS can cause spatial disorganization of endothelial cells and degeneration and structural fragility of the aneurysmal wall (23, 24). Moreover, atherogenic and proinflammatory signal pathways may be activated in endothelial cells under low WSS, which can predispose the aneurysmal wall to thinning and rupture (25, 26).

Other risk factors (such as hypertension and age) associated with aneurysm rupture may also affect the aneurysmal hemodynamic via cerebral vasculature remodeling. Hypertension could significantly augment the increase in vessel length and tortuosity, which make the vessel more vulnerable to flow-induced damage (27). Moreover, Jeon et al. found that hypertension was a significant predictive factor for aneurysm growth (28). Zhang et al. demonstrated that with increase of patient age, cerebral artery bifurcation angle was significantly increased and changes of bifurcation angle were associated with significant hemodynamic stress alterations (29).

To help physicians assess aneurysmal rupture risk and guide clinical decision making, several rupture predictions models that integrate multiple conventional rupture risk factors have been created. The PHASES score is one of these models that has been widely studied. The PHASES score was developed by pooling data from six large longitudinal aneurysm studies and provides the 5-year absolute risk of aneurysm rupture based on 6 risk factors (population, hypertension, age, size of aneurysm, earlier subarachnoid hemorrhage from another aneurysm, site of aneurysm) (10). It reflect the trends of aneurysm rupture risk and has been applied in some studies. Bijlenga et al. found that PHAESE scores of stable UIA patients were significantly lower than high risk of rupture group (30). Backes reported that higher PHASES scores were associated with an increased risk of aneurysm growth and aneurysm growth has a strong association with aneurysm rupture (31). However, the accuracy, sensitivity, and specificity of the PHASES score have been criticized (30). Several recent studies have reported that aneurysms in patients with a low PHASES score are still associated with a non-negligible likelihood of rupture (32, 33). One reason may be that the prevalence of patients with a low PHASES score is high, thus most instances of aneurysmal subarachnoid hemorrhage come from these patients with a low PHASES score. The other reason may be that the PHASES scoring system does not include one or more factors that are important predictors of aneurysm rupture, such as hemodynamic factors. A multidimensional prediction model that includes important morphological and hemodynamic parameters may lead to a better assessment of aneurysmal rupture risk.

This study has several limitations. First, it was retrospective in design and included patients from only one center. Second, we used several assumptions (rigid wall, laminar flow, and Newtonian blood), which might have introduced bias. Third, the PHASES score is not the gold standard to identify patients with UIA at high risk of rupture, although it is widely accepted. Fourth, only patients with a solitary intracranial aneurysm were included; therefore, our findings may not be applicable to patients with multiple aneurysms. Finally, the sample size was limited and future prospective multicenter studies with a large cohort are warranted to validate our results.

## Conclusion

Low WSS was significantly associated with higher risk of intracranial aneurysm rupture as determined by PHASES score, indicating that hemodynamics may play an important role in aneurysmal rupture. In the future, a multidimensional rupture risk prediction model that includes hemodynamic parameters should be investigated.

## Data Availability Statement

The raw data supporting the conclusions of this article will be made available by the authors, without undue reservation.

## Ethics Statement

The studies involving human participants were reviewed and approved by the Ethics Committee of Zhujiang Hospital of Southern Medical University. Written informed consent to participate in this study was provided by the participants' legal guardian/next of kin.

## Author Contributions

ZT performed the statistical analysis and the manuscript writing. XL interpreted the data. CW and XF were responsible for aneurysmal model reconstruction and analyzed. KS, YT, and HS acquired the data. CD and XY conceived and designed the research. All authors contributed to the article and approved the submitted version.

## Funding

This work was funded by the National Key Research Development Program (Grant Numbers: 2016YFC1300804 and 2016YFC1300800), the Science and Technology Project Foundation of Guangdong province (Grant Number: 2016A020215098), the Key Project of Clinical Research of Southern Medical University (Grant Number: LC2016ZD024), and the Guangdong Provincial Clinical Medical Centre for Neurosurgery (Grant Number: 2013B020400005).

## Conflict of Interest

The authors declare that the research was conducted in the absence of any commercial or financial relationships that could be construed as a potential conflict of interest.

## Publisher's Note

All claims expressed in this article are solely those of the authors and do not necessarily represent those of their affiliated organizations, or those of the publisher, the editors and the reviewers. Any product that may be evaluated in this article, or claim that may be made by its manufacturer, is not guaranteed or endorsed by the publisher.
